# Child development and nutritional status in 12–59 months of age in resource limited setting of Ethiopia

**DOI:** 10.1186/s41043-020-00214-x

**Published:** 2020-04-14

**Authors:** Shimelash Bitew Workie, Tesfa Mekonen, Tefera Chane Mekonen, Wubalem Fekadu

**Affiliations:** 1 College of Health Sciences and Medicine, Wolaita Sodo University, Wolaita Sodo, Ethiopia; 2 College of Medicine and Health Sciences, Bahir Dar University, Bahir Dar, Ethiopia; 3 College of Medicine and Health Sciences, Wollo University, Dessie, Ethiopia

**Keywords:** Child development, Development delay, Ages and stages questionnaire

## Abstract

**Background:**

Early years of life are period of maximal growth and development of human brain. Development of young child is influenced by biological endowment and health of child, nutritional status of child, relationships with primary caregivers, family, and support systems in the community. This study was aimed to assess childhood development in relation to their nutritional status.

**Method:**

Community-based cross-sectional study was employed. Multi–stage systematic random sampling technique was used to select 626 children aged 12-59 months with mother/caregivers’ pairs in Wolaita district in 2015. Child development assessment was done using third edition of age and stage questionnaire. Height and weight were measured by trained data collectors then the WHO Anthro version 3.2.2 software was used to convert nutritional data indices. Data were entered into Epi-info version 3.3.5 and was exported and analyzed using STATA version 14. Correlation and multiple logistic regression were used.

**Result:**

High risk of developmental problem in children were 19.0% with 95% CI (16.06%, 22.3%), and it is expressed as communication 5.8%, gross motor 6.1%, fine motor 4.0%, personal social 8.8%, and problem solving 4.1%. One-third (34.1%) of the study participants were stunted while 6.9% and 11.9% of them were wasted and underweight respectively. Weight-for-age (WAZ) and height-for-age positively correlated with all five domains of development, i.e., with communication, gross motor, fine motor, personal social, and problem solving (*r* = 0.1 − 0.23; *p* < 0.0001, and *r* = 0.131 − 0.249; *p* < 0.0001) respectively.

**Conclusion and recommendation:**

Overall child development was directly related with nutritional status. So, available resources should be offered to decrease children undernutrition. Further assessment on childhood development of children is necessary

## Introduction

Early childhood period is the most important developmental phase in life. The term “child development” indicates advancement of the child in all areas of human functioning: social and emotional, cognitive, communication, and movement [1, 2]. Development of child is a maturational process resulting in an ordered progression of perceptual, motor, cognitive, language, socio emotional, and self-regulation skills. Multiple factors influence the acquisition of competencies and skills, including health, nutrition, security and safety, responsive care giving, and early learning [3].

Childhood under nutrition is contributing to childhood morbidity, mortality, impaired intellectual development, suboptimal adult work capacity, and increased risk of diseases in adulthood; hence it is one of the major global health problems [4, 5]. It can be existed in the form of wasting (acute malnutrition, weight-for-height Z-score), stunting (chronic malnutrition, height-for-age Z-score), or underweight (weight-for-age Z-score) [4, 6].

The 2016 Ethiopian Demographic and Health Survey (EDHS) showed that there has been improvement in the nutritional status of children in the past 15 years. Stunting was 38% in 2016 EDHS all over Ethiopia while severe stunting was 18%. Similarly, 24% of children under age five are underweight and 7% are severely underweight. However, there is no change in the prevalence of wasting, as it remained about 10% and 2% are severely wasted [7]. The Government of Ethiopia has continued its commitment to nutrition by developing the second phase of the National Nutrition Program (NNP II, 2016-2020) [8].

Despite long experience in fighting childhood illness and mortality, health care providers in low and middle income countries face new challenges in promoting child development. Early childhood development in developing countries estimated that over 200 million children in developing countries are not reaching their full developmental potential [9]. Developmental difficulties during early childhood is increasingly recognized in low and middle income countries as important contributors to morbidity in children and adults. Child development of the cognitive, social-emotional, and language and movement functions is influenced by the biological endowment and health of the child, as well as by the relationships with the primary caregivers, family, and support systems in the community. The early years of life are a period of maximal growth and development of the human brain and are therefore extremely important in determining whether the person reaches his or her full potential [1]. Hence, this study was designed to determine the relationship between childhood development and their nutritional status, and result obtained may be used by policy makers and program managers in different parts of the country.

## Methods and materials

### Study design and setting

A community-based cross-sectional study was conducted from children residing in Wolaita zone from May 2015-June 30, 2015. Wolaita zone is found in SNNPR region covering an area of 4471.3 km^2^. It is located at 380 km South of Addis Ababa and 157 km away from Hawassa town. For administrative purpose, it is divided into twelve woredas and three administrative cities. Total population of the zone is estimated about 1,721,339 with a density of 385 inhabitants per square kilometer. Wolaita Sodo town is the administrative center of the zone.

### Sample size determination and sampling procedure

All children 12-59 months of age residing in Wolaita zone were the source population, whereas all children residing in selected kebeles were considered as the study population. Sample size was determined by single population proportion formula by considering 44.1% prevalence of stunting in SNNPR from EDHS 2011 [10], margin of error 5%, confidence level of 95%, design effect of 1.5, and 10% of non-respondent, and then the final sample size was found 626**.** Multi–stage systematic sampling was used to select the study participants. First, 3 woredas and 2 town administrations were selected from 12 districts and 3 town administration. Boloso Sore, Sodo Zuriya, Offa woredas, Areka town, and Sodo town were selected by lottery method. Then sample size was allocated based on proportionate of the under-five population in each woreda. One urban and three rural kebeles were selected based on lottery method. Households which have children 12-59 months were selected using systematic sampling by taking the sampling frame from health extension workers.

### Variables

Dependent variable childhood development score of children by age and stage questionnaire version three.

The primary independent variables were child nutritional variables (weight-for-height, height-for-age, and weight-for-age). The other independent variables were residence, formal education , wealth status, age of the mother, immunization of child, birth order of the child, sex of the child, age of the child, initiation of complementary feeding, dietary diversity score, meal frequency score, place of delivery, term of delivery, and others. Formal education of the mother is categorized as yes if a woman had attended any governmental formal education. Wealth status was defined as high, medium, and low (poor) based on principal component analysis. Ever breast feed, food frequency, term of delivery, dietary diversity score, and initiation of complementary feeding were defined as per different literature [11, 12].

### Measurement and data collection procedure

Pre-tested interview administer questionnaire was used for socio-demographic, household economic status variables, nutritional variables, maternal variables, child health related factors, and food accesses at household variables. This pre-tested questionnaire was developed after reviewing different literatures [10, 13, 14]. Child development assessment was done using the third edition of age and stage questionnaire (ASQ-3) of mental development. The ASQ-3 has five subscales: communication, gross motor, fine motor, problem solving, and personal-social. Age and stage questionnaire was answered as “yes” scored as 10, “sometimes” scored 5, and “not at all” scored 0 [15]. Each form contains 30 items, six for each subscale, written in a simple language. Some questions are specific for certain age groups, while other items are used for a wider age range and are repeated in the different age-specific questionnaires. The ages and stages questionnaire has validity of 0.83-0.88, reliability of 0.90-0.94, sensitivity of 38-91%, and specificity of 79.3-91% [16–18]. Child development was measured at their dwelling as per the recommendation of ASQ-3. Each domain was classified into three (high risk for developmental, needs monitoring and well development) for each age category based on ASQ-3. Finally, child development was categorized as developmental delay and well development based on recommendation.

Every child was examined medical status on their dwelling by supervisors and data collectors. Pre-test was conducted on 5% of the total sample size in one of the town administrations and the surrounding rural area which have similar basic socio-economic characteristics as the study kebeles, and necessary correction were made. Data were collected from caregivers or mothers of the children by ten BSc holder nurses who could communicate well with the local language.

Anthropometric data were taken by supervisors. Supervisors were 4 and had master’s degree in public health and had health background. Anthropometric data was collected following the WHO standards. Children dietary frequency score and dietary diversity was assessed based on the last 24-h recall method. Dietary diversity score was assessed based on IYCF recommendation among 7 food categories [11].

Data collectors and supervisors were trained for 3 days and a regular supervision with practical session for height and weight measurements were done. Technical error of measurement (TEM) was computed during training. For this, an expert was taken two measurements weight and height of ten children and let supervisors take measurements of all ten children twice. Then, data entered and computed by the ENA SMART software and was confirmed as the result generated was acceptable.

For age and stage questionnaire training was given by psychiatrists who is knowledgeable and experienced on the age and stage questionnaire and by principal investigators.

In addition, regular check-up for completeness and consistency of the data was made daily. Moreover, high emphasis was given in designing data collection instruments for its simplicity and reproducibility. Weights and heights were measured twice, and the mean values were used for the analysis. Standardization anthropometric measurements were conducted to see whether the data collectors have good precision and accuracy and fortunately the precision and the accuracy of most of the enumerators were acceptable.

### Data management and analysis

Pre-coded data were entered to Epi info version 3.5.3 and the WHO Anthro software was used to convert nutritional data into Z-scores of indices by using the new WHO growth standard. Children whose height-for-age, weight-for-height, and weight-for-age < − 2 SD from the median of the reference population were considered stunted, wasted, and underweight respectively. Then, data were exported to the STATA software version 14 for data processing and analysis. Principal component analysis was done using household assets possession to construct wealth index, as a proxy measure of household socio-economic status. Household socioeconomic status was finally divided into terciles (rich, medium, and poor). Assumptions of principal component analysis were checked. Relation between childhood development and their nutritional status was assessed with Pearson correlation coefficient with *P* value. Multiple logistic regression was used to assess factors associated with child nutrition and mental development, and *P* value of less than 0.05 will be considered significant.

## Result

### General characteristics of the population

A total of 605 (96.8%) children with their mothers/caregivers were interviewed. From total respondents 413 (68.26%) were rural kebele residence. Nearly 91% of mothers were married and 69.26% (419) mothers had attended formal education. Mean age of mothers was 27.25, with SD of 6.025 and a minimum of 15 years and maximum of 50 years. On the average, 5 people lived at the household with SD, 1.5 and 46% of the children were living with a total family size of less than 4 (Table 1).

**Table 1:** Socio demographic characteristics of study participants in Wolaita District, Ethiopia, 2015 (*N* = 605)

Variable		Residence		Total (frequency (%))
		Urban (frequency (%))	Rural (frequency (%))	
Total family	2-4	73 (38.02)	211 (51.09)	284 (46.9)
	5-7	94 (48.96)	172 (41.65)	266 (44.0)
	> = 8	25 (13.02)	30 (7.26)	55 (9.1)
Head of household	Mother	42 (21.88)	90 (21.79)	132 (21.82)
	Father	150 (78.13)	323 (78.21)	473 (78.18)
Marital status of the mother	Single	7 (3.65)	10 (2.42)	17 (2.8)
	Married	169 (88.02)	379 (91.77)	548 (90.6)
	Divorced	8 (4.17)	16 (3.87)	24 (4.0)
	Widowed	8 (4.17)	8 (1.94)	16 (2.6)
Maternal formal education	No	23 (11.86)	163 (39.58)	186 (30.74)
	Yes	169 (85.14)	250 (60.42)	419 (69.26)
Mother occupational status	Housewife	139 (72.40)	268 (64.89)	407 (67.3)
	Merchant	30 (15.63)	66 (15.98)	104 (17.2)
	Government civil servant	3 (1.56)	40 (9.69)	43 (7.1)
	Farmer	7 (3.65)	12 (2.91)	19 (3.1)
	Student	4 (2.08)	8 (1.94)	12 (2.0)
	Day laborer	8 (4.17)	12 (2.91)	20 (3.3)
Mothers’ age	Young (15-24)	44 (22.91)	138 (33.41)	182 (30.1)
	Middle (25-34)	116 (60.42)	225 (54.49)	341 (56.4)
	Late (> = 35)	32 (16.67)	50 (12.1)	82 (13.6)
Wealth status	High status	68 (35.42)	138 (33.41)	211 (34.9)
	Medium status	60 (31.25)	137 (33.17)	190 (31.4)
	Low status (poor)	64 (33.33)	138 (33.41)	204 (33.7)

From total children, 307 (50.7%) were males. Mean age of children were 33.87 months (SD 13.9 month). Above half of the children were toddlers. Thirty seven percent of children were first child for their mothers. Almost all children were term at delivery which is 99.3% and sixty percent (60.5%) of mothers were delivered at government health facility. Out of the total children, 95.5% of them were fully immunized. Twenty-three percent of children were sick in the last 2 weeks before survey (Table 2).

**Table 2:** Child socio-demographic, feeding and health status living in Wolaita District, South Ethiopia, 2015 (*N* = 605)

Variable		Residence		Total (frequency (%))
		Urban (frequency (%))	Rural (frequency (%))	
Sex of the child	Male	96 (50.0)	211 (51.09)	307 (50.7)
	Female	96 (50.0)	202 (48.51)	298 (49.3)
Age of the child	Toddler (1-3 years)	90 (46.88)	223 (53.00)	313 (51.74)
	Preschool (3-5 years)	102 (52.12)	190 (47.00)	292 (48.26)
Order of birth for the child	First child	45 (23.44)	179 (43.34)	224 (37.0)
	Second	53 (27.6)	99 (23.97)	152 (25.1)
	Third and forth	52 (27.08)	88 (21.31)	140 (23.1)
	Above 5th	42 (21.88)	47 (11.38)	89 (14.7)
Ever breast feed	Yes	188 (97.92)	411 (99.51)	599 (99.00)
	No	4 (2.08)	2 (0.49)	6 (1.00)
Time to initiating breast feed from delivery (*N* = 599)	Within 1 h	108 (56.84)	290 (70.39)	398 (66.11)
	Above 24 h	21 (11.06)	26 (6.31)	47 (7.81)
	Within 24 h	61 (32.11)	96 (23.30)	157 (26.08)
Currently on Breast Feeding (*N* = 599)	Yes	69 (36.51)	165 (40.24)	234 (39.07)
	No	120 (63.49)	245 (59.76)	365 (60.93)
Initiation of complementary feeding (*N* = 599)	Early initiation (< 6 months)	3 (1.59)	19 (4.63)	22 (3.67)
	At 6 months	151 (79.89)	341 (83.17)	492 (82.14)
	Late initiation (> 6 months)	35 (18.52)	50 (12.20)	85 (14.19)
Was child sick in the past 2 weeks	Yes	52 (27.08)	91 (22.03)	143 (23.6)
	No	140 (72.92)	322 (77.97)	462 (76.4)
Fully immunized	Yes	179 (93.23)	399 (96.61)	578 (95.5)
	No	13 (6.77)	14 (3.39)	27 (4.5)
Medical problem of child	Yes	3 (1.56)	3 (0.73)	6 (1.0)
	No	189 (98.44)	410 (99.27)	599 (99.0)
History of accident	Yes	2 (1.04)	6 (1.45)	8 (1.3)
	No	190 (98.96)	407 (98.55)	597 (98.68)
Place of delivery	Home with relatives	81 (42.19)	91 (22.03)	172 (28.4)
	Home with traditional birth attendant	26 (13.54)	19 (4.6)	45 (7.4)
	Government health facility	83 (43.23)	283 (68.52)	366 (60.5)
	Private health facility	2 (1.04)	20 (4.84)	22 (3.6)
Type of delivery (*N* = 388)	Spontaneous vaginal delivery (SVD)	68 (80.00)	258 (85.71)	326 (84.46)
	Cesarean section (CS)	6 (7.06)	17 (5.65)	23 (5.96)
	Instrumental delivery	10 (11.76)	26 (8.64)	36 (9.33)
Was the child term at delivery	Yes	190 (98.96)	411 (99.52)	601 (99.34)
	No	2 (1.04)	2 (0.48)	4 (0.66)
Maternal medical complication	Yes	23 (11.98)	38 (9.20)	61 (10.08)
	No	169 (88.02)	375 (90.80)	544 (89.92)
Anti-natal care (ANC) follow up	Yes	141 (73.44)	351 (84.99)	492 (81.3)
	No	51 (26.56)	62 (15.01)	113 (18.68)
Dietary food frequency	> = 3	107 (55.73)	331 (80.15)	438 (72.4)
	< 3	85 (44.27)	82 (19.85)	167 (27.6)
Dietary diversity score	> = 4	79 (41.15)	282 (68.28)	361 (59.67)
	< 4	113 (58.85)	131 (31.72)	244 (40.33)

#### Nutritional status and dietary practices of the children

Dietary frequency score of 24-h recall method shows almost all (72.4%) of the children were above and equal to minimum recommendation. Fifty-nine percent of children diversity score were less than 4 types (Table 2).

Prevalence of stunting, wasting, and underweight were 34.1% with 95% CI (30.4-37.9%), 6.9% with 95% CI (5.2-9.3%), and 11.9% with 95% CI (9.5-14.7%) respectively. Prevalence of severely stunted, severely wasted, and severely underweight children were 15.6%, 3%, and 4.1% respectively (Z-score < − 3SD). No overweight or obese cases were observed (Table 3).

**Table 3:** Nutritional status of children by age of the children in Wolaita District, South Ethiopia, 2015

Nutritional parameter		Age category	
	Toddler (1-3 years) (*N* = 313) (%)	Preschool (3-5 years) (*N* = 292) (%)	*N* = 605 (%)
Stunted (HAZ < − 2)	36.1	31.85	34.1
Severely stunted (HAZ < − 3)	18.2	13.7	15.6
Underweight (WAZ < − 2)	10.5	13.4	11.9
Severely underweight (WAZ < − 3)	3.87	4.3	4.1
Wasted (WHZ < − 2)	7.7	6.2	6.9
Severely wasted (WHZ < − 3)	3.6	1.7	3

### Child developmental status

Mean ASQ-3 score for total score was 231.23, with standard deviation from 61.54, all with a range from zero to 300. The mean ASQ-3 score for each domain was range from 39.05-53.28, with standard deviation from 13.19-19.28, all with a range from zero to 60. This study revealed that high risk of developmental problem in children were 19.0% with 95% CI (16.06%, 22.3%) and it is expressed as communication 5.8%, gross motor 6.1%, fine motor 4.0%, personal social 8.8%, and problem solving 4.1%. From communication 14.7%, from gross motor 9.6%, from fine motor 12.6%, from personal social 17.9% and from problem solving 6.9% were needs monitoring. The rest were well developed according to their age (Table 4).

**Table 4:** Child developmental status of children by age of the children in Wolaita District, South Ethiopia, 2015

Type of development domain	Mean (SD)	High risk for developmental delay	Needs monitoring	Well development
Communication	48.28 (15.6)	5.8% (35)	14.7% (89)	79.5% (481)
Gross motor	53.28 (13.19)	6.1% (37)	9.6% (58)	84.3% (510)
Fine motor	39.06 (19.28)	4.0% (24)	12.6% (76)	83.5% (505)
Personal social	40.89 (18.29)	4.1% (25)	17.9% (108)	78.0% (472)
Problem solving	49.72 (14.79)	8.8% (88)	6.9% (42)	84.3% (510)

#### Relationship between nutritional status and child development

Height-for-age (stunting) Z-score had significant association with communication, gross motor, fine motor, personal social, and problem solving. Weight-for-age (stunting) Z-score had significant association with communication, gross motor, fine motor, personal social, and problem solving (Table 5).

**Table 5:** Relation between child developmental status and nutritional status of children in Wolaita District, South Ethiopia, 2015

Variables	Child development domains				
	Communication *r* (*P* value)	Gross motor *r* (*P* value)	Fine motor *r* (*P* value)	Personal social *r* (*P* value)	Problem solving *r* (*P* value)
Weight-for-height (WHZ)	− 0.019 (0.639)	0.042 (0.3000)	0.065 (0.112)	0.058 (0.158)	− 0.08 (0.851)
Height-for-age [19]	0.207 (0.000)**	0.263 (0.000)**	0.147 (0.000)**	0.159 (0.000)**	0.186 (0.000)**
Weight-for -age (WAZ)	0.125 (0.002)*	0.227 (0.000)**	0.159 (0.000)**	0.16 (0.000)**	0.110 (0.007)*

*Significant at *P* value < 0.05

**Significant at *P* value < 0.0001

#### Factors affecting child cognitive development

First childhood development was categorized as risk for developmental delay and normal development. In bivariate logistic regression being stunted, underweight, dietary diversity score, mother’s age at birth, frequency of feeding, immunization status, starting month for complementary feeding, birth order of child, residence, and educational status of the mother were significantly associated with developmental status of the child.

Multiple logistic regression analysis showed that children who are stunted, starting month for complementary feeding, who had no get minimum dietary diversity score, and birth order of the child were associated with developmental delay. As the childbirth order increased by 1 being developmentally delayed increased by 1.2 times with 95% CI (1.01, 1.34). Stunted children had 2.2 times more likely to be developmentally delayed with 95% CI (1.3-3.5). Children who started complimentary feeding below and above 6 months of age were associated with developmental delay compared with those started at age of 6 months with AOR 3.73, 95% CI (1.5-8.8) and 3.24, 95% CI (1.87-5.62), respectively. Children who get minimum dietary diversity score below 4 were 2.1 times more likely to be developmentally delayed with 95% CI (1.3-3.4) (Table 6).

**Table 6:** Relation between child developmental status and nutritional status of children in Wolaita District, South Ethiopia, 2015

Variable		Developmental delay		COR (95% CI)	AOR (95% CI)
		Normal	Delayed		
Residence	Rural	346	67	1	
	Urban	144	48	0.58 (0.38, 0.88)	
Mother formal education	No	123	63	1	
	Yes	367	52	0.62 (0.39-0.99)	
Order of child				1.2 (1.1-1.33)	1.2 (1.1-1.3)
Mothers age at birth of index child	Young (14-24)	152	30	1	
	Middle (25-34)	280	61	1.1 (0.68-1.78)	
	Late (> = 35)	58	24	2.0 (1.13-3.88)	
Immunization status	Yes	473	105	1	
	No	17	10	2.64 (1.17-5.95)	
Initiation of complementary feeding	Early initiation (< 6 months)	11	11	3.69 (1.66-8.22)	3.73 (1.5-8.8)
	At 6 months	419	73	1	1
	Late initiation (> 6 months)	54	31	3.16 (1.9-5.2)	3.24 (1.8-5.6)
Dietary diversity score	> = 4	315	46	1	1
	< 4	175	69	2.7 (1.8-4.1)	2.1 (1.3-3.4)
Meal frequency	> = 3	369	69	1	
	< 3	121	46	2.0 (1.3-3.1)	
Stunted	No	344	55	1	1
	Yes	146	60	2.6 (1.7-3.9)	2.2 (1.3-3.5)
Underweight	No	443	90	1	
	Yes	47	25	2.6 (1.5-4.5)	

## Discussion

In this survey, one-third (34.1%) of the study participants were stunted while 6.9% and 11.9% of them were wasted and underweight respectively. Generally according to the WHO’s classification, the prevalence of stunting in the study area was very high. It is almost similar with that of EDHS 2016 national and regional prevalence which was 38% and 38.6% respectively, but it is lower than Amhara regional (46%) [7]. As compared to other small pocket studies in different parts of Ethiopia, prevalence of stunting in the study area was found to be low. For example, prevalence in Haromaya district [14], Bule Hora district [20], northwest Ethiopia Dembia district [21], and northeast Ethiopia Lalibela [22] was 45.8%, 47.6%, 46%, and 47.3 respectively, while it is almost in line with finding from Hossana town [23] was 35.4%. This might be due to difference in method used, sample size variation, variation in agro-ecological pattern, and feeding practices.

In this survey, high risk of developmental problem in children were 19.0% with 95% CI (16.06%, 22.3%) and it is expressed as communication 5.8%, gross motor 6.1%, fine motor 4.0%, personal social 8.8%, and problem solving 4.1%. This finding was lower compared to the lancet 2016 report for the world which was 43 percent of children under 5 years of age—an estimated 250 million—living in low- and middle-income countries are at risk of suboptimal development [3]. This finding was in line with the finding from Iran which was done in children from 4-60 months of age [24].

Height-for-age and weight-for-age of children had significant correlation with communication, gross motor, fine motor, personal social, and problem solving both in correlation. This finding is similar with the finding from Sidama, Ethiopia. There is a significant difference in mean cognitive test scores between stunted and non-stunted, and between underweight and normal-weight children [25]. But in multivariable logistic regression analysis, we find stunting is the only determinate for child development. This is in line with study conducted in Vietnam, Ethiopia, and Peru [26, 27]. Height-for-age Z-score was linearly associated with cognitive, communication, and motor development Z-scores by study done in Tanzania [28].

Weight-for-age (stunting) Z-score had significant association with communication, gross motor, fine motor, personal social, and problem solving with correlation analysis. But underweight had no influence on the development in multivariable binary logistic regression.

According to this survey, factors that affect childhood development are birth order of the child, time for initiation of complementary feeding, and dietary diversity score. As birth order increase by 1, developmental delay increased by 20%. This is not similar with study conducted in Vietnam, Ethiopia, and Peru [26]. This might be that this study has done limited setting.

Time for initiation of complementary feeding had significant association with child development. Those children who started complementary feeding below the age of 6 month and above 6 month had significant effect on child development.

Children who get minimum dietary diversity score below 4 were 2.1 times more likely to be developmentally delayed. This is similar with finding from Goba, Ethiopia [29].

The other important health parameters were very significantly better compared to national and regional estimates. Almost all were vaccinated fully according to their age. These findings were above the national estimate [30].

The limitation of this study was conducted cross sectional design due to it measure exposure and outcome at the same time. It does not show cause and effect association. It would be better if the study design is a follow-up study. We measure mental development at one time, but one time measurement might lead to biased. It would be better if it measured repeatedly than one time.

## Conclusion and recommendation

Childhood developmental delay is a major public health problem in resource limited countries. Childhood stunting also remains a major public health challenge in Ethiopia. Childhood developmental delay had significant relation with stunting and underweight. Factors that increase the risk of developmental delay were stunted, birth order, time for initiation complementary feeding, and minimum dietary score. Thus, nutritional intervention program in Wolaita district, Ethiopia should focus on these factors for decrease developmental delay.

## Data Availability

The datasets during and/or analyzed during the current study is available from the corresponding author on reasonable reques.

## Abbreviations

AOR: Adjusted odds ratio
CI: Confidence interval
COR: Crude odds ratio
EDHS: Ethiopian Demographic and Health Survey
FANTA: Food and Nutrition Technical Assistance Project
IYCF: Infant and young child feeding
SNNPR: Southern Nation National and People Region
WHO: World Health Organization
